# Paternal environment effects are driven by female reproductive fluid but not sperm age in an external fertilizer

**DOI:** 10.1098/rsbl.2023.0368

**Published:** 2023-11-22

**Authors:** Jessica H. Hadlow, Rowan A. Lymbery, Jonathan P. Evans

**Affiliations:** Centre for Evolutionary Biology, School of Biological Sciences, The University of Western Australia, Crawley, Western Australia 6009, Australia

**Keywords:** parental effect, cryptic female choice, sperm selection, gamete interactions, non-genetic inheritance

## Abstract

Sperm ageing after ejaculation can generate paternal environment effects that impact offspring fitness. In many species, female reproductive fluids (FRFs), i.e. ancillary fluids released by eggs or within the female reproductive tract, may protect sperm from ageing and can additionally interact with sperm to influence offspring viability. This raises the intriguing prospect that FRFs may alleviate paternal effects associated with sperm ageing. Here, we test this novel hypothesis using the broadcast spawning mussel, *Mytilus galloprovincialis*. We show that incubating sperm in FRF prior to fertilization increases offspring viability, and that these effects occur independently of sperm age. Our results provide novel evidence that FRFs allow females to selectively bias fertilization toward higher quality sperm within an ejaculate, which in turn yields more viable offspring. We consider this FRF-mediated paternal effect in the context of female physiological control over fertilization and the transgenerational effects of female-regulated haploid selection.

## 1. Introduction

There is substantial evidence that variation in the paternal environment can influence offspring phenotypes due to changes to the ejaculate [1–3]. Inheritance due to such paternal environment effects can occur, for example, via epigenetic alterations to sperm or non-sperm ejaculate components [4,5], or due to selection on haploid genetic variation among sperm cells within an ejaculate [6]. To date, most research on paternal environment effects has focused on male germline exposure to environmental stressors prior to ejaculate release, i.e. while sperm are developing or stored in male reproductive organs [3]. However, the post-ejaculation environment, whether external or within female reproductive tracts, can also influence sperm in ways that have implications for future generations (e.g. [7,8]; see [3,5,9]).

Post-meiotic sperm age is a key determinant of reproductive fitness in many species and can also generate paternal environment effects that influence offspring performance [10–12]. Post-meiotic ageing comprises both pre-ejaculatory ageing (after spermatogenesis but during male sperm storage) and post-ejaculatory ageing (between sperm release and fertilization) [10]. In the latter case, ejaculated sperm can exhibit reductions in motility and fertilization capacity with age ([8,13,14], but see [15,16]). Such effects may result from ATP depletion, or an accumulation of reactive oxygen species (ROS) due to oxidative stress [10,11,17], and can be exacerbated by increases in sperm metabolism caused by sperm dilution, i.e. the respiratory dilution effect [18]. Moreover, post-ejaculatory ageing of sperm can impact offspring, although the effects remain equivocal (e.g. positive effect: [19], negative effect: [20], no effect: [13]).

Females are expected to evolve strategies to mitigate sperm ageing costs [21]. These include remating frequently to acquire fresh sperm [22], preferentially storing young sperm [23], or possessing sperm storage organs that reduce ageing (e.g. by lowering sperm metabolism and reducing ROS production) [24,25]. Females also typically produce reproductive fluids (female reproductive fluids; FRFs), derived from their reproductive organs or eggs, that can counteract sperm ageing [26]. For example, FRFs ‘rescue' aged sperm by enhancing fitness-determining phenotypes, including sperm motility [27,28] and sperm viability [29]. Moreover, recent research has demonstrated that FRFs, independent of paternal or maternal genotype, can influence embryonic phenotypes, thus revealing previously unforeseen indirect parental effects on offspring viability [30]. This finding, coupled with the positive effects of FRF on aged sperm, suggests that FRF may also moderate paternal environment effects associated with sperm age.

Here, we explore the effects of both post-ejaculatory sperm ageing and FRF exposure on fertilization success and offspring survival. Our study uses a powerful within-ejaculate design and the broadcast spawning blue mussel, *Mytilus galloprovincialis*, a system in which gamete-mediated parental effects have recently been reported [30,31]. We use established methods to manipulate the presence of egg-derived FRF during fertilization [30,32], and exploit dilution effects on sperm metabolism to experimentally age sperm through controlled dilutions [33]. In *M. galloprovincialis*, FRF typically improves sperm motility (e.g. [33,34]) and ultimately influences fertilization success [34–36] and offspring viability [30,34], though the effect of FRF on fertilization and offspring viability depends on the identity of the sperm, egg, and FRF donors [30,34]. With this system, we ask if post-ejaculatory sperm age reduces (i) fertilization success and (ii) offspring survival, and we test the hypothesis that female reproductive fluids can alleviate potential fitness reductions, e.g. fertilization or offspring effects, resulting from sperm age.

## 2. Material and methods

We collected mussels from Woodman Point, Western Australia (32°14′03.6″ S, 115°76′25″ E) from August to October 2022. On collection days, we induced spawning by exposing mussels to filtered seawater heated to 26°C [35]. We used standard procedures to estimate and adjust gamete concentrations (e.g. [34,35]; see electronic supplementary material).

### (a) Experimental overview

Our design allowed us to test for main and interacting effects of sperm age and FRFs on fertilization and offspring survival. We ran the experiment in logistically feasible blocks, each comprising 3–6 males, and used a split-ejaculate design with two treatments, each with two levels (see electronic supplementary material, figure S1). A detailed description of our experimental procedures can be found in the electronic supplementary material. In brief, for each experimental replicate we split ejaculates from each male into four subsamples that were each exposed to one of four treatment combinations. First, we used an ageing treatment, whereby sperm were diluted either 1 h prior to fertilization trials (aged) or immediately prior to fertilization (fresh). This treatment exploits the accelerated ageing sperm experience upon dilution (the respiratory dilution effect; [18]; refer to the electronic supplementary material for further details). Second, we briefly exposed aged and fresh sperm samples to (i) seawater containing FRFs (seawater in which eggs had been suspended to release chemical cues), or (ii) seawater only. In each block, FRF was collected from 5–6 females using standard procedures prior to treating sperm ([32,34]; electronic supplementary material). Sperm were mixed with the exposure treatment (i.e. FRF or seawater) immediately prior to fertilization trials. We then added treated ejaculate subsamples from each individual focal male to pooled eggs from the same group of females used to collect FRF. To minimize possible effects of FRF from the fertilization egg pools, we filtered these eggs immediately before adding treated sperm [30,32]. Differences between FRF and seawater treatments can therefore be attributed to whether sperm were transiently exposed to FRF prior to fertilization.

Fertilized eggs were left to develop for 1.5 h until a multi-cell stage, at which point we removed two subsamples from each egg pool (see the electronic supplementary material). We fixed one subsample for measuring fertilization rates and diluted another in a vial of seawater for estimating offspring survival 48 h later. The egg pools were gently mixed prior to taking subsamples, which ensured samples contained comparable numbers of eggs. We measured fertilization success by counting the number of eggs with polar bodies or undergoing cell division out of 100 haphazardly sampled eggs. To estimate offspring survival we counted surviving larvae in 50 µl aliquots (number of live larvae ranged from 24 to 88). Fertilization and offspring counts were performed blind to treatment. Across a total of eight blocks, we collected data from *n* = 40 males tested across each treatment combination for a total of 160 assays for both fertilization and offspring viability trials.

### (b) Data analysis

All analyses were conducted in R, version 4.1.1 [37]. Initial data exploration revealed two outliers (datapoints more than 1.5 x interquartile range outside of the upper and lower quartiles) for fertilization success, and six outlier datapoints for offspring survival. Inclusion or exclusion of these points from subsequent analyses did not qualitatively change our conclusions (electronic supplementary material, tables S1–S2), but we nevertheless report the more conservative analyses excluding outliers (which included 158 and 154 observations across *n* = 40 males for fertilization success and offspring survival, respectively) in the main text.

We modelled fertilization rate and offspring survival with generalized linear mixed models using ‘glmmTMB' [38]. Fertilization rate was modelled with a binomial error distribution and logit link function. We included fixed effects of sperm age (aged or fresh), exposure treatment (FRF or seawater), and their interaction, a random intercept term for male ID to account for repeated measures, and random intercepts for block ID to account for possible among-block variation. We modelled the number of surviving offspring with a Poisson error distribution and log link function, the same fixed and random effects as in the fertilization model, and an additional fixed covariate of fertilization rate to control for variation in offspring numbers caused by differences in fertilization success [30]. Model assumptions were checked with ‘DHARMa' [39] and testing of fixed effects was determined with Type III Wald *χ*^2^ tests in the package ‘car' [40]. Standardized fixed effect sizes and 95% confidence intervals (CIs) were calculated with the ‘effectsize' package [41], and presented on the latent scale. Where interaction effects were significant (i.e. slopes for the effect of one factor differed across levels of the other), we performed *post hoc* contrasts to test for differences in the effect of FRF within each level of sperm age in the package ‘emmeans' [42]. To support the contrasts we provide approximated effect sizes (*d*)*,* and 95% CIs, converted from *post hoc* test statistics (*t*) [41]. We additionally used ‘emmeans' to calculate estimated marginal means and standard errors for plotting [42].

## 3. Results

**Figure 1.**
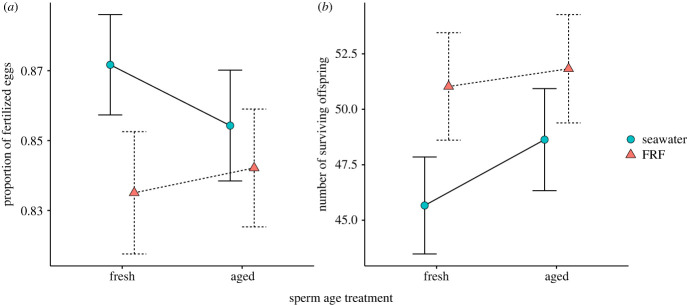
Effect of sperm age treatment (aged or fresh) and sperm exposure treatment (FRF: red triangles, dashed lines; seawater: blue circles, solid lines) on (*a*) fertilization rate and (*b*) offspring survival. Dots and whiskers represent estimated marginal means ± standard errors.

Fertilization rates differed between FRF treatments, i.e. whether sperm were exposed to FRF or seawater immediately prior to fertilization ($\chi_{1}^{2}=20.81$, *p* < 0.001, effect size = −0.29, CIs = −0.42, −0.17), and between aged and fresh sperm ($\chi_{1}^{2}=5.15$, *p* = 0.023, effect size = −0.15, CIs = −0.27, −0.02). The interaction between sperm age and exposure treatments also impacted fertilization ($\chi_{1}^{2}=4.99$, *p* = 0.025, effect size = 0.20, CIs = 0.02, 0.38; figure 1*a*). *Post hoc* contrasts revealed these effects were driven by differences within the fresh treatment, with fertilization success greater when sperm were exposed to seawater only than when exposed to FRF (*t*_152_ = 4.56, *p* < 0.001, *d* = 0.37, CIs = 0.21, 0.53; electronic supplementary material, table S3). Within the aged treatment, fertilization rates did not differ among sperm exposed to seawater only or FRF (*t*_152_ = 1.52, *p* = 0.131, *d* = 0.12, CIs = −0.04, 0.28; electronic supplementary material, table S3). Offspring survival was greater when sperm were added to FRF than when exposed to seawater only ($\chi_{1}^{2}=11.10$, *p* < 0.001, effect size = 0.11, CIs = 0.05, 0.18; figure 1*b*) and was positively associated with the covariate for fertilization rate ($\chi_{1}^{2}=7.09$, *p* = 0.008, effect size = 0.05, CIs = 0.01, 0.09; electronic supplementary material, figure S1). Offspring survival was not significantly affected by an interaction between age and exposure treatments ($\chi_{1}^{2}=1.08$, *p* = 0.299, effect size = −0.05, CIs = −0.14, 0.04), or by sperm age ($\chi_{1}^{2}=3.66$, *p* = 0.056, effect size = 0.06, CIs = 0.00, 0.13).

## 4. Discussion

We report the novel finding that offspring survival is higher when sperm are exposed to FRF than seawater, irrespective of sperm age. This is despite an interaction between sperm age and sperm exposure to FRF impacting fertilization success, exposing fresh, but not aged, sperm to FRF reduced fertilization rates compared to when sperm were exposed to seawater only. Taken together, these findings lead us to propose that FRF allows females to selectively bias fertilization towards a subset of sperm, which subsequently yields more viable offspring. We discuss this finding in detail below. We then consider our original hypothesis that FRF can mitigate sperm age-related paternal environment effects and the likely influence of haploid selection on our results.

Adding sperm to FRF resulted in greater offspring survival than when sperm were treated with seawater only. Remarkably, this effect was evident after just a brief incubation period, as sperm were added to fertilization pools immediately after dilution in FRF. There are at least two possible non-mutually exclusive explanations for this finding. First, adding FRF to sperm may have altered sperm cells in a way that impacts offspring phenotypes. In many taxa, including *M. galloprovincialis* [43,44], FRFs cause physiological changes to sperm in preparation for fertilization and these could have transgenerational effects [30]. For example, in mammals, the molecular and structural alterations that occur to sperm during sperm capacitation in mammals, e.g. changes in RNA expression, may be transmissible to offspring [4,9,45]. This explanation could partially explain our results, but if it were the sole mechanism at play, we might expect high fertilization rates to be maintained in the FRF treatments, which was not the case. A second, exciting possibility is that FRFs could act as a selective filter to favour a subset of sperm within an ejaculate, allowing eggs to select sperm that produce higher quality offspring [46]. It is well-established that FRFs enable females to selectively target ejaculates from *different* competing males (e.g. based on genetic compatibility [28,32,35]). Our findings may point to even more nuanced mechanisms that further refine the female's capacity to select optimal sperm for fertilization. Intriguingly, a recent study demonstrated that ovarian fluid in zebrafish selects sperm with better DNA integrity and viability from *within* a single ejaculate [47]. Studies designed to further explore the transgenerational implications of within-ejaculate cryptic female choice via FRF will be useful to test this second scenario [48].

Fertilization rates were lower in FRF-exposed treatments than in seawater-only treatments, although the effect is small and only manifested for fresh sperm. This finding was unexpected, given fitness-related sperm traits are typically improved by FRF, both in *M. galloprovincialis* [33,34] and many other taxa [26,28,29]. One possible explanation for this finding is that rapid energy loss resulting from sperm hyperactivation led to overall lower fertilization in our FRF treatment (e.g. [14]). In support of this, sperm motility in *M. galloprovincialis* declines at a faster rate upon exposure to FRF than seawater only [33]. A study of two broadcast spawning invertebrates also attributed fertilization declines to waning sperm energy reserves after sperm incubation in FRF [14]. However, this idea does not account for the lack of an FRF effect on fertilization when sperm were aged. An alternate explanation, which follows the ideas discussed above for our offspring fitness results, is that FRF imposes haploid selection for high quality sperm within an ejaculate [49,50]. This would explain why only a subset of fresh sperm (presumably those that produce higher quality offspring) achieved success in the FRF treatment. For the aged sperm, however, the selective filtering effect of FRF at fertilization may have been reduced if short-lived sperm were already removed from the fertilization pool by the ageing treatment. Although we would require further testing to confirm the occurrence of haploid selection via FRF (e.g. selection on genetic variation within an ejaculate), the combination of our fertilization and offspring survival results are consistent with FRF allowing females to bias fertilization to a specific set of sperm that produce more viable offspring.

We predicted that FRFs would alleviate fitness reductions caused by sperm age, but ultimately found no evidence for deleterious effects of post-ejaculatory sperm age on offspring survival. Instead, there was a trend in the opposite direction, whereby offspring survival tended to increase with sperm age. As we note above, reports of post-ejaculatory sperm age effects on offspring in the literature are equivocal (e.g. [13,19,20]), although beneficial effects of sperm age on offspring have been previously described. For example, haploid selection for longer-lived sperm improves embryo survival in zebrafish [50], ascidian embryos sired by longer-lived sperm hatch faster than those sired by shorter-lived sperm [19], and variation in sperm longevity impacts offspring development in Atlantic salmon [51]. It is plausible that our ageing treatment exposed within-ejaculate variation in sperm quality to haploid selection but we did not have enough statistical power to detect the effect. Additionally, the effect of FRF on fertilization success was only apparent in fresh sperm, and there does appear to be a trend towards a larger beneficial effect of FRF on offspring in the fresh sperm treatment, which would be consistent with ageing doing some of the selective ‘work' of removing poor-quality sperm. However, further tests are needed to confirm these ideas. Overall, our data are consistent with the idea that haploid (sexual) selection imposed by FRF has a greater effect on offspring than post-ejaculatory sperm age.

## Data Availability

Data and code associated with this manuscript are available from the Dryad Digital Repository: https://doi.org/10.5061/dryad.d51c5b08c [52]. Electronic supplementary material is available online [53].

## References

[1] Crean AJ, Bonduriansky R. 2014 What is a paternal effect? Trends Ecol. Evol. **29**, 554-559. (10.1016/j.tree.2014.07.009) 25130305

[2] Bonduriansky R, Day T. 2018 Extended heredity. Oxford, UK: Princeton University Press.

[3] Evans JP, Wilson AJ, Pilastro A, Garcia-Gonzalez F. 2019 Ejaculate-mediated paternal effects: evidence, mechanisms and evolutionary implications. Reproduction **157**, R109-R126. (10.1530/rep-18-0524) 30668523

[4] Immler S. 2018 The sperm factor: paternal impact beyond genes. Heredity **121**, 239-247. (10.1038/s41437-018-0111-0) 29959427 PMC6082889

[5] Crean AJ, Immler S. 2021 Evolutionary consequences of environmental effects on gamete performance. Philos. Trans. R. Soc. B **376**, 20200122. (10.1098/rstb.2020.0122) PMC8059621 33866815

[6] Immler S. 2019 Haploid selection in ‘diploid’ organisms. Annu. Rev. Ecol. Evol. Syst. **50**, 219-236. (10.1146/annurev-ecolsys-110218-024709)

[7] Ritchie H, Marshall DJ. 2013 Fertilisation is not a new beginning: sperm environment affects offspring developmental success. J. Exp. Biol. **216**, 3104-3109. (10.1242/jeb.087221) 23661780

[8] Gasparini C, Daymond E, Evans JP. 2018 Extreme fertilization bias towards freshly inseminated sperm in a species exhibiting prolonged female sperm storage. R. Soc. Open Sci. **5**, 172195. (10.1098/rsos.172195) 29657813 PMC5882737

[9] Pitnick S, Wolfner MF, Dorus S. 2020 Post-ejaculatory modifications to sperm (PEMS). Biol. Rev. **95**, 365-392. (10.1111/brv.12569) 31737992 PMC7643048

[10] Pizzari T, Dean R, Pacey A, Moore H, Bonsall MB. 2008 The evolutionary ecology of pre- and post-meiotic sperm senescence. Trends Ecol. Evol. **23**, 131-140. (10.1016/j.tree.2007.12.003) 18280006

[11] Reinhardt K. 2007 Evolutionary consequences of sperm cell aging. Q. Rev. Biol. **82**, 375-393. (10.1086/522811) 18217528

[12] Reinhardt K, Dobler R, Abbott J. 2015 An ecology of sperm: sperm diversification by natural selection. Annu. Rev. Ecol. Evol. Syst. **46**, 435-459. (10.1146/annurev-ecolsys-120213-091611)

[13] Hotzy C, Xuhui B, Larva T, Immler S. 2020 Intrinsic post-ejaculation sperm ageing does not affect offspring fitness in Atlantic salmon. J. Evol. Biol. **33**, 576-583. (10.1111/jeb.13590) 31961980

[14] Williams ME, Bentley MG. 2002 Fertilization success in marine invertebrates: the influence of gamete age. Biol. Bull. **202**, 34-42. (10.2307/1543220) 11842013

[15] Vega-Trejo R, Fox RJ, Iglesias-Carrasco M, Head ML, Jennions MD. 2019 The effects of male age, sperm age and mating history on ejaculate senescence. Funct. Ecol. **33**, 1267-1279. (10.1111/1365-2435.13305)

[16] Meunier L, Sorci G, Abi Hussein H, Hingrat Y, Rehspringer N, Saint-Jalme M, Lesobre L, Torres Carreira J. 2022 Pre-but not post-meiotic senescence affects sperm quality and reproductive success in the North African houbara bustard. Front. Ecol. Evol. **10**, 1195. (10.3389/fevo.2022.977184)

[17] Aitken RJ, Drevet JR, Moazamian A, Gharagozloo P. 2022 Male infertility and oxidative stress: a focus on the underlying mechanisms. Antioxidants **11**, 306. (10.3390/antiox11020306) 35204189 PMC8868102

[18] Chia FS, Bickell LR. 1983 Echinodermata. In Reproductive biology of marine invertebrates volume II: spermatogenesis and sperm function (eds KG Adiyodi, RG Adiyodi), pp. 545-620. New York, NY: John Wiley and Sons.

[19] Crean AJ, Dwyer JM, Marshall DJ. 2012 Fertilization is not a new beginning: the relationship between sperm longevity and offspring performance. PLoS ONE **7**, e49167. (10.1371/journal.pone.0049167) 23155458 PMC3498328

[20] Wagner RH, Helfenstein F, Danchin E. 2004 Female choice of young sperm in a genetically monogamous bird. Proc. R. Soc. Lond. B **271**, S134-S137. (10.1098/rsbl.2003.0142) PMC1810032 15252964

[21] Siva-Jothy MT. 2000 The young sperm gambit. Ecol. Lett. **3**, 172-174. (10.1046/j.1461-0248.2000.00146.x)

[22] Turnell BR, Reinhardt K. 2022 Sperm metabolic rate predicts female mating frequency across *Drosophila* species. Evolution **76**, 573-584. (10.1111/evo.14435) 35064568

[23] Reinhardt K, Siva-Jothy MT. 2005 An advantage for young sperm in the house cricket *Acheta domesticus*. Am. Nat. **165**, 718-723. (10.1086/430010) 15937751

[24] Paynter E, Millar AH, Welch M, Baer-Imhoof B, Cao D, Baer B. 2017 Insights into the molecular basis of long-term storage and survival of sperm in the honeybee (*Apis mellifera*). Sci. Rep. **7**, 40236. (10.1038/srep40236) 28091518 PMC5238380

[25] Ribou A-C, Reinhardt K. 2012 Reduced metabolic rate and oxygen radicals production in stored insect sperm. Proc. R. Soc. B **279**, 2196-2203. (10.1098/rspb.2011.2422) PMC3321705 22279170

[26] Gasparini C, Pilastro A, Evans JP. 2020 The role of female reproductive fluid in sperm competition. Phil. Trans. R. Soc. B **375**, 20200077. (10.1098/rstb.2020.0077) 33070736 PMC7661459

[27] Elofsson H, Mcallister BG, Kime DE, Mayer I, Borg B. 2003 Long lasting stickleback sperm; is ovarian fluid a key to success in fresh water? J. Fish Biol. **63**, 240-253. (10.1046/j.1095-8649.2003.00153.x)

[28] Poli F, Immler S, Gasparini C. 2019 Effects of ovarian fluid on sperm traits and its implications for cryptic female choice in zebrafish. Behav. Ecol. **30**, 1298-1305. (10.1093/beheco/arz077)

[29] Gasparini C, Evans JP. 2013 Ovarian fluid mediates the temporal decline in sperm viability in a fish with sperm storage. PLoS ONE **8**, e64431. (10.1371/journal.pone.0064431) 23691216 PMC3653924

[30] Lymbery RA, Berson JD, Evans JP. 2020 Indirect parental effects on offspring viability by egg-derived fluids in an external fertilizer. Proc. R. Soc. B **287**, 20202538. (10.1098/rspb.2020.2538) PMC7739940 33290674

[31] Lymbery RA, Kennington WJ, Evans JP. 2021 The thermal environment of sperm affects offspring success: a test of the anticipatory paternal effects hypothesis in the blue mussel. Biol. Lett. **17**, 20210213. (10.1098/rsbl.2021.0213) 34228940 PMC8260270

[32] Lymbery RA, Kennington WJ, Evans JP. 2017 Egg chemoattractants moderate intraspecific sperm competition. Evol. Lett. **1**, 317-327. (10.1002/evl3.34) 30283659 PMC6121861

[33] Hadlow JH, Evans JP, Lymbery RA. 2023 Female reproductive fluids ‘rescue’ sperm from phenotypic ageing in an external fertilizer. Proc. R. Soc. B **290**, 20230574. (10.1098/rspb.2023.0574) PMC10206448 37221848

[34] Oliver M, Evans JP. 2014 Chemically moderated gamete preferences predict offspring fitness in a broadcast spawning invertebrate. Proc. R. Soc. B **281**, 20140148. (10.1098/rspb.2014.0148) PMC4043089 24741014

[35] Evans JP, Garcia-Gonzalez F, Almbro M, Robinson O, Fitzpatrick JL. 2012 Assessing the potential for egg chemoattractants to mediate sexual selection in a broadcast spawning marine invertebrate. Proc. R. Soc. B **279**, 2855-2861. (10.1098/rspb.2012.0181) PMC3367782 22438495

[36] Hadlow JH, Evans JP, Lymbery RA. 2020 Egg-induced changes to sperm phenotypes shape patterns of multivariate selection on ejaculates. J. Evol. Biol. **33**, 797-807. (10.1111/jeb.13611) 32125748

[37] R Core Team. 2021 R: a language and environment for statistical computing. Vienna, Austria: R Foundation for Statistical Computing. http://www.r-project.org.

[38] Brooks ME, Kristensen K, Van Benthem KJ, Magnusson A, Berg CW, Nielsen A, Skaug HJ, Machler M, Bolker BM. 2017 glmmTMB balances speed and flexibility among packages for zero-inflated generalized linear mixed modeling. R J. **9**, 378. (10.32614/RJ-2017-066)

[39] Hartig F. 2022 DHARMa: residual diagnostics for hierarchical (multi-level/mixed) regression models. See https://cran.r-project.org/package=DHARMa.

[40] Fox J, Weisberg S. 2019 An R companion to applied regression, 3rd edn. Thousand Oaks, CA: Sage. See https://socialsciences.mcmaster.ca/jfox/Books/Companion/.

[41] Ben-Shachar M, Lüdecke D, Makowski D. 2020 effectsize: Estimation of effect size indices and standardized parameters. J. Open Source Softw. **5**, 2815. (10.21105/joss.02815)

[42] Lenth RV. 2021 emmeans: Estimated marginal means, aka least-squares means. See https://cran.r-project.org/package=emmeans.

[43] Kekäläinen J, Larma I, Linden M, Evans JP. 2015 Lectin staining and flow cytometry reveals female-induced sperm acrosome reaction and surface carbohydrate reorganization. Sci. Rep. **5**, 15321. (10.1038/srep15321) 26470849 PMC4607886

[44] Kekäläinen J, Evans JP. 2017 Female-induced remote regulation of sperm physiology may provide opportunities for gamete-level mate choice. Evolution **71**, 238-248. (10.1111/evo.13141) 27921298

[45] Li Y et al. 2018 High throughput small RNA and transcriptome sequencing reveal capacitation-related microRNAs and mRNA in boar sperm. BMC Genomics **19**, 736. (10.1186/s12864-018-5132-9) 30305024 PMC6180635

[46] Firman RC. 2018 Postmating sexual conflict and female control over fertilization during gamete interaction. Ann. N. Y. Acad. Sci. **1422**, 48-64. (10.1111/nyas.13635) 29524360

[47] Cattelan S, Devigili A, Santacà M, Gasparini C. 2023 Female reproductive fluid attracts more and better sperm: implications for within-ejaculate cryptic female choice. Biol. Lett. **19**, 20230063. (10.1098/rsbl.2023.0063) 37340806 PMC10282568

[48] Kekäläinen J. 2022 Cryptic female choice within individual males—a neglected component of the postmating sexual selection? J. Evol. Biol. **35**, 1407-1413. (10.1111/jeb.14081) 35988118 PMC9804180

[49] Immler S, Otto SP. 2018 The evolutionary consequences of selection at the haploid gametic stage. Am. Nat. **192**, 241-249. (10.1086/698483) 30016160

[50] Alavioon G, Hotzy C, Nakhro K, Rudolf S, Scofield DG, Zajitschek S, Maklakov AA, Immler S. 2017 Haploid selection within a single ejaculate increases offspring fitness. Proc. Natl Acad. Sci. USA **114**, 8053-8058. (10.1073/pnas.1705601114) 28698378 PMC5544320

[51] Immler S, Hotzy C, Alavioon G, Petersson E, Arnqvist G. 2014 Sperm variation within a single ejaculate affects offspring development in Atlantic salmon. Biol. Lett. **10**, 20131040. (10.1098/rsbl.2013.1040) 24522632 PMC3949374

[52] Hadlow JH, Lymbery RA, Evans JP. 2023 Data from: Paternal environment effects are driven by female reproductive fluid but not sperm age in an external fertilizer. *Dryad Digital Repository*. (10.5061/dryad.d51c5b08c) PMC10664279 37991195

[53] Hadlow JH, Lymbery RA, Evans JP. 2023 Paternal environment effects are driven by female reproductive fluids but not sperm age in an external fertilizer. Figshare. (10.6084/m9.figshare.c.6927482) PMC10664279 37991195

